# A novel method for the isolation of single cells mimicking circulating tumour cells adhered on Smart Bio Surface slides by Laser Capture Microdissection

**DOI:** 10.1371/journal.pone.0297739

**Published:** 2024-03-08

**Authors:** Grazia Visci, Doron Tolomeo, Angelo Lonoce, Aram Arshadi, Lorenzo Bascetta, Gianluca Trotta, Margot van Riel, Joris Robert Vermeesch, Roberta Carbone, Clelia Tiziana Storlazzi

**Affiliations:** 1 Department of Biosciences, Biotechnology and Environment, University of Bari Aldo Moro, Bari, Italy; 2 Tethis S.p.a., Milan, Italy; 3 Institute of Intelligent Industrial Technologies and Systems for Advanced Manufacturing, Consiglio Nazionale delle Ricerche, Bari, Italy; 4 Department of Human Genetics, KU Leuven, Leuven, Belgium; Penn State Health Milton S Hershey Medical Center, UNITED STATES

## Abstract

In recent years, the importance of isolating single cells from blood circulation for several applications, such as non-invasive tumour diagnosis, the monitoring of minimal residual disease, and the analysis of circulating fetal cells for prenatal diagnosis, urged the need to set up innovative methods. For such applications, different methods were developed. All show some weaknesses, especially a limited sensitivity, and specificity. Here we present a new method for isolating a single or a limited number of cells adhered to SBS slides (Tethis S.p.a.) (a glass slide coated with Nanostructured Titanium Dioxide) by Laser Capture Microdissection (LCM) and subsequent Whole Genome Amplification. SBS slides have been shown to have an optimal performance in immobilizing circulating tumour cells (CTCs) from early breast cancer patients. In this work, we spiked cancer cells in blood samples to mimic CTCs. By defining laser parameters to cut intact samples, we were able to isolate genetically intact single cells. We demonstrate that SBS slides are optimally suited for isolating cells using LCM and that this method provides high-quality DNA, ideal for gene-specific assays such as PCR and Sanger sequencing for mutation analysis.

## 1. Introduction

Genomic instability is a hallmark of cancer cells [1], implying the occurrence of various structural and numerical genetic mutations and resulting in the genesis of diverse tumour cell sub-populations (tumour heterogeneity) [2, 3]. A portion of cancer cells is shed from the primary solid tumour into the blood vessels after epithelial-mesenchymal transition (EMT) resulting in circulating tumour cells (CTCs). Those cells can survive in the peripheral vascular system and initiate the development of metastases. Because of their essential role in the metastatic process, CTCs have been investigated as diagnostic, prognostic, and predictive biomarkers in many types of cancer. They have been used to enable early detection of tumour lesions (primary and metastatic), to identify minimal residual disease and to stratify patients, based on the detection of therapeutic targets or resistance mechanisms [4].

Cancer cell heterogeneity represents a significant challenge for targeted therapies in the clinical management of patients [5]. In this context, the isolation of CTCs is crucial for the non-invasive molecular analysis of cancer cells in patients.

To date, several technologies have been developed to isolate and characterize CTCs from blood. Those methods are usually based on the enrichment and/or depletion of CTCs using specific antibodies, filtration by size, or separation by microfluidic systems [2]. These methods lay their foundations on the biological and physical features of CTCs [5]. However, CTCs isolation remains a major challenge because of two main issues: i) CTCs are extremely rare in the blood vessels (0–1,000 CTCs among 5 billion erythrocytes and 10 million leukocytes per mL of blood) [6]; ii) the detection of CTCs is technically demanding. In principle, a perfect approach would imply the analysis of an “unmanipulated” clinical sample to minimize the risk of CTC loss.

The last years have seen considerable innovation in instrumentation and analytical techniques for single-cell isolation. One of these is the Laser Capture Microdissection (LCM) technology [7]. LCM is an ideal tool for rapidly collecting a large number of single cells. It can precisely target and capture cells of interest, despite tissue heterogeneity. LCM contact-free isolation and separation allows precision and avoid contamination [8]. This method is quick and versatile, used in various applications, including the molecular characterization of cancer cells. Also, it offers the possibility of identifying specific cells by labelling them with fluorescent antibodies [9].

The present work describes a new strategy for isolating and analysing single cells mimicking CTCs by combining the LCM technology with Whole Genome Amplification (WGA). Smart BioSurface (SBS) slides were used to optimize the yield of CTC recovery from blood. These slides are capable to efficiently immobilize living and non-spontaneously adhering cells, such as cells deriving from blood, enabling a comprehensive capture approach to liquid biopsy [10]. Cluster-assembled TiO_2_ films result from a random stacking of nanoparticles and are characterized, at the nanoscale, by granularity and porosity mimicking extracellular matrix structures [11]. Previous results indicated that cluster-assembled nanostructured TiO_2_ is a biocompatible surface for the cell adhesion of different kinds of normal and cancer cells [12].

To validate our method, we mimicked CTCs in liquid biopsy-like samples by mixing cancer cell line cells with white blood cells (WBCs) from healthy donors. We chose two cancer cell lines (PANC-1 and SW-620) as representatives of the most devastating tumour types (pancreas and colon carcinoma, respectively) and focused on specific hotspot mutations in frequently mutated genes (Table 1). The samples were dispensed on SBS slides, and the cancer cells, identified by immuno-staining, were selectively isolated by LCM. We obtained a high-efficiency recovery of single cancer cells by LCM and an effective amplification of single-cell DNA by Whole Genome Amplification, as evaluated by its Quality Control. The efficacy of the method consists of the possibility to perform downstream molecular analyses at the single-cell level, such as identifying specific nucleotide mutations. Hence, the present methodology could be offered as a potential non-invasive approach for identifying and capturing CTCs from the blood of cancer patients. Interestingly, the present methodology could also be applied for other purposes, such as isolating circulating fetal cells for non-invasive prenatal diagnosis.

**Table 1:** Cell lines and mutations investigated to validate the proposed cell isolation method.

Cell line	Nucleotide mutation	Zygosity status
PANC-1	TP53 c.818G>A (p.Arg273His)	Homozygous
SW-620	KRAS 35G>A (G12D)	Homozygous

## 2. Materials and methods

The protocol described in this peer-reviewed article is published on protocols.io, dx.doi.org/10.17504/protocols.io.81wgbx5kolpk/v1, and is included for printing as S1 File with this article.

The protocol is subdivided into seven sections. The first two sections–sections 1 (Preparation of spiked-in samples) and 2 (Cell lines selection and culturing)—describe how we obtained SBS slides from samples mimicking blood specimens with CTCs. We prepared these slides to validate our protocol; in future applications, sections 1–2 can be replaced with the most suitable approaches, depending on the purpose of the experiment, such as the preparation of peripheral blood smears to isolate CTCs from cancer patients. The subsequent three sections–section 3 (Mimicking circulating tumor cell (CTC) detection by Immuno-staining), 4 (Giemsa staining), and 5 (Isolation of mimicking CTCs by Laser Capture Microdissection (LCM))–outline how to identify mimicking CTCs on slides, export their microscope coordinates, and use them to isolate the single cells by microdissection after Giemsa staining. In section 3, the Immuno-staining can be performed with different antibodies or substituted with other techniques to identify the cells of interest. The final two sections– 6 (WGA and Quality control (QC)) and 7 (Polymerase chain reaction (PCR) and Sanger sequencing)–detail all steps to amplify and use single-cell DNA for subsequent molecular analyses (section 6), such as mutation detection (section 7).

### 2.1 Preparation of spiked-in samples

Healthy donor blood was purchased from Biopredic International (Parc d’affaires, Saint Gregoire, France), through anonymous healthy volunteers, after discarding the first 3 mL of blood draw to minimize keratinocyte contamination. Blood samples were processed with red blood cell lysis through ammonium chloride buffer (0.15M NH_4_Cl, 9.93mM KHCO_3_, 0.13mM EDTA, Sigma-Aldrich, St. Louis, Missouri, USA). WBCs were then separated by centrifugation (400×g, 5 min at RT), resuspended in DPBS (Sigma-Aldrich), and counted using Trypan blue (Sigma-Aldrich). A predefined number of cells (50–100 cells for each slide) from selected cancer cell lines (see paragraph 2.2) was then spiked into WBCs pellet. The cell mix was dispensed on SBS slides, patented by Tethis S.p.A (Milan, Italy), at the optimal concentration of 2.5 x 10^6^ cells per slide [10]. Subsequently, the slides were fixed with 4% paraformaldehyde (PFA) (Sigma-Aldrich) and stored at -80°C.

### 2.2 Cell lines selection and culturing

To validate our method, we chose two cancer cell lines harbouring known point mutations in homozygous state (see Table 1), i.e., the pancreatic cancer PANC-1 and the colon cancer SW-620. These cell lines and the BEAS-2B normal bronchial epithelial cell line were obtained from the American Type Culture Collection (ATCC, Manassas, VA, USA). BEAS-2B was used as WGA positive control (see paragraph 2.6). PANC-1 was cultured in DMEM medium (EuroClone, Pero, Milan, Italy), while SW-620 and BEAS-2B in RPMI-1640 medium (EuroClone), both supplemented with 10% fetal bovine serum (EuroClone) and Penicillin-Streptomycin mixture (Sigma-Aldrich). All cells were incubated at 37°C and 5% CO_2_. For spike-in and WGA experiments, cells were gently detached, resuspended in DPBS (Sigma-Aldrich), and counted with Trypan blue (Sigma-Aldrich). Genomic DNA was extracted from BEAS-2B cells using the gSYNC^TM^ DNA Extraction Kit (Geneaid Biotech Ltd., New Taipei City, Taiwan) according to the manufacturer’s instructions and measured using the Qubit™ Fluorometer (Life Technologies, Carlsbad, USA).

### 2.3 Mimicking CTC detection by immuno-staining

Slides prepared with samples obtained by mixing cell line cells with WBCs were permeabilized with methanol ice-cold (Sigma-Aldrich) and then blocked in a solution of 5% Normal Goat Serum (Cell Signalling, Danvers Massachusetts, United States, product code: 5425). Immuno-staining was performed with an anti-CD45 pan-leukocyte marker (Bio-Rad Laboratories, Inc., CA, USA, product code: MCA87) and an anti-pan-cytokeratins antibody (Sigma-Aldrich, product code: C2562). Nuclei were counterstained with Hoechst (ThermoFisher Scientific, Waltham, Massachusetts, USA).

After staining, slides were scanned through the automated fluorescence microscopy Metasystems platform (ZEISS AxioImagerZ2 microscope) and analysed using Tethis proprietary imaging algorithms [10]. Cancer cell line cells (mimicking CTCs) were manually selected by trained operators following the criteria of staining positivity to pan-CKs and negativity to CD45. The position of each mimicking CTC was also identified using slide coordinates, which were appropriately transferred to the LCM microscope.

### 2.4 Giemsa staining

To be visualized at the LCM microscope, cells were Giemsa stained. The slide was soaked in a Coplin jar containing PBS 1X for 5 minutes to facilitate the cover slide removal. After gently removing the cover slide, the slide was air dried in a vertical position and stained by adding 1mL of 7.4% Giemsa stain (ThermoFisher Scientific) for 20 minutes. Then, it was washed by briefly dipping in and out of a Coplin jar containing distilled water (one or two dips). Finally, the slide was air-dried vertically and observed under the microscope.

### 2.5 Isolation of mimicking CTCs by LCM

The LCM was carried out using the Leica Microsystems LMD6500 platform (Leica Biosystems, Wetzlar, Germany), according to the manufacturer’s instructions, but setting up appropriate laser parameters (see paragraph 3.1). We searched for the mimicking CTCs at a 10X magnification by exploiting the previously identified slide coordinates (as described in paragraph 2.3). The LCM was then performed at a 63X magnification: first, the WBCs around the mimicking CTC were microdissected into a ‘trash’ collection cap of a 0.2 mL DNase-free PCR tube. Subsequently, we microdissected the single cell of interest into a new cap filled with 30 μL of a Lysis Buffer (0.5% NP40, Proteinase K 0.67 μg/μL, 0.032 M Tris-HCl pH 8) to prevent the risk of sample loss. After visualizing the single cell into the cap, we unloaded the tube holder, carefully recovered the PCR tube, and closed it. For negative controls, 30 μL of Lysis Buffer was added to an empty PCR tube cap. The collected samples were incubated at 50°C overnight to allow the cells to lysis (without overturning the tubes). After the incubation, the samples were spun down in a centrifuge for 10 minutes at maximum speed and stored at 4°C until further processing.

### 2.6 WGA and Quality control (QC)

DNA amplification was performed using the Ampli1™ WGA Kit (Menarini Silicon Biosystems, Castel Maggiore, Italy) since it allows genome amplification from single fixed cells, as reported in the manufacturer’s instructions.

Since the WGA requires a minimal sample volume of 1 μL, we let the samples evaporate using a heater at 50°C, then proceed with WGA, following the manufacturer’s instructions.

Briefly, samples underwent a second lysis step with a 2 μL lysis reaction mix (42°C, overnight). DNA was digested by adding 2 μL of digestion reaction mix and incubating at 37°C for 5 minutes. Following enzyme inactivation (65°C for 5 minutes), 5 μL of ligation mix was added, and the pre-annealed adapter nucleotides were ligated to the fragmented DNA at 15°C for 1 hour. Finally, the Primary PCR Reaction Mix (40 μL) was added to each sample to obtain the DNA amplification.

To assess the performance of the Ampli1™ method, each reaction run included negative and positive controls. The negative (not template) control, consisting of 1 μL of H_2_O, was added to exclude possible contamination during WGA. We used two different positive controls. The first consists of a pool of five BEAS-2B cells (either fixed or not with 4% of PFA); the second corresponds to approximately 30 pg of BEAS-2B genomic DNA. These control samples allowed us to evaluate the performance of our method regardless of cell isolation, cell lysis, and DNA extraction/amplification efficiency.

DNA concentration of the WGA products was measured using the Qubit™ Fluorometer.

The quality of WGA products was checked by performing a multiplex-PCR using the Ampli1™ QC kit (Menarini Silicon Biosystems). BEAS-2B genomic DNA (100 ng) was chosen as a positive control. According to the manufacturer’s instructions, products with at least one/two out of four PCR products were suitable for gene-specific analyses (e.g., for detecting specific point mutations). Three/four PCR products indicate that the samples could be used for successful genome-wide studies (metaphase/array CGH). Samples were checked by agarose gel electrophoresis (2% agarose, Condalab, Madrid, Spain), stained with GelRed™ (Biotium, Landing Pkwy, Fremont, USA), and visualized on the ChemiDoc^TM^ MP Imaging System (Bio-Rad Laboratories).

### 2.7 Polymerase chain reaction (PCR) and Sanger sequencing

We performed PCR assays to specifically amplify genetic regions harbouring the selected point mutations in each cell line used to validate the method. The primer pairs (Table 2) were designed using Primer 3 plus (https://www.bioinformatics.nl/cgi-bin/primer3plus/primer3plus.cgi) according to the Ampli1™ WGA Kit manufacturer’s recommendations. PCR experiments, Sanger sequencing, and sequence analyses were performed as previously described [13].

**Table 2:** Primers used to amplify mutated genomic regions in microdissected cells.

Primer name	Sequence	Map position (GRCh38/hg38)	PCR product size (bp)
KRAS_ex2_For	TCATTATTTTTATTATAAGGCCTGCTG	chr12:25,245,388–25,245,414	151
KRAS_ ex2_Rev	CAAGATTTACCTCTATTGTTGGATCA	chr12:25,245,264–25,245,289	
TP53_ex4_For	GGGACAGGTAGGACCTGATTT	chr17:7,673,877–7,673,897	152
TP53_ex4_Rev	TCTTGCGGAGATTCTCTTCC	chr17:7,673,746–7,673,765	

## 3. Results

### 3.1 Laser beam parameters setup for an efficient microdissection capture of single cells from SBS slides

**Fig 1:**
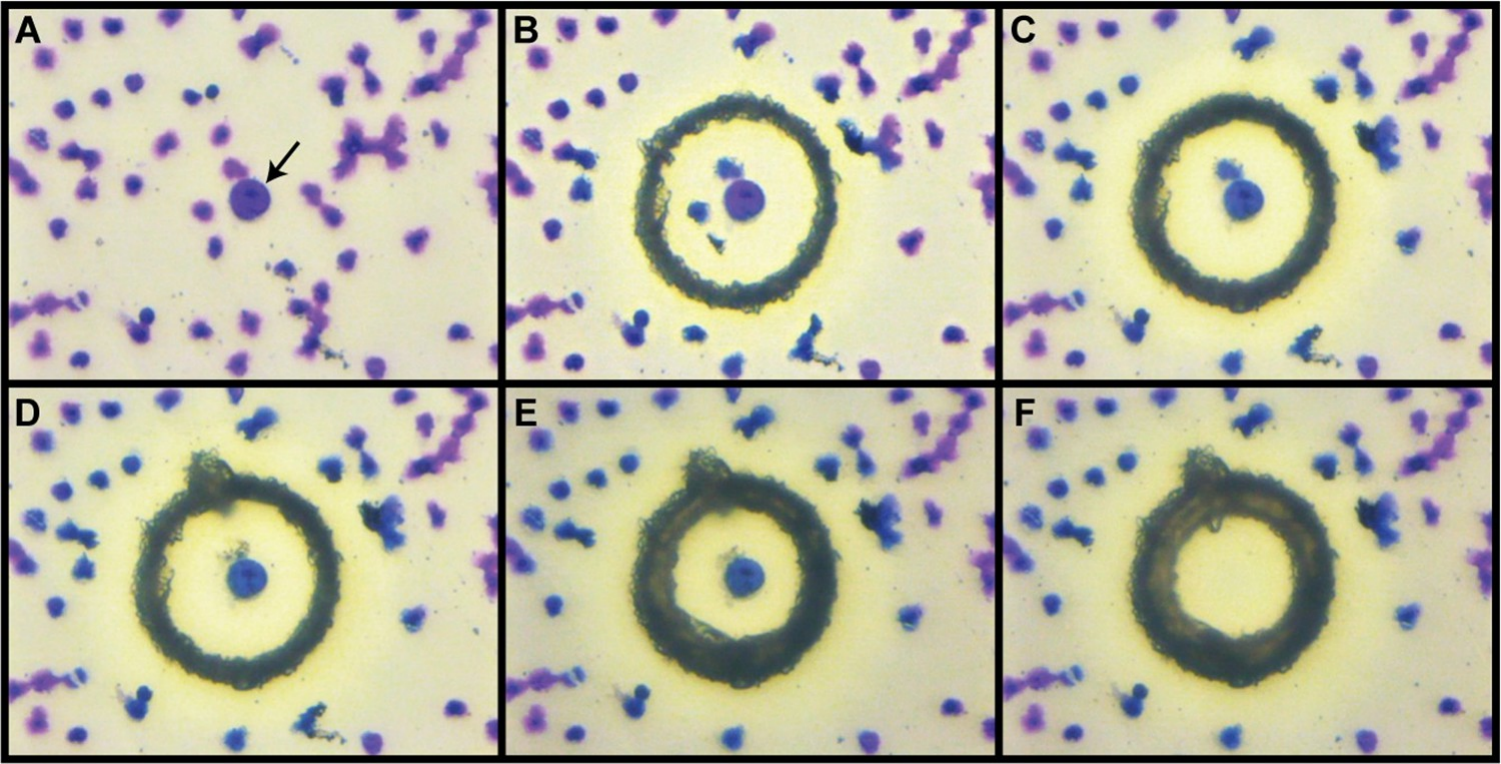
Single-cell laser microdissection capture procedure from SBS slides. After visualizing the cell of interest at a 63X magnification (black arrow) (A), and discarding the other cells around it in a ‘trash’ cap (B, C, D), the laser microdissection of the sample is performed by moving the laser cut line close to the cell of interest until it falls into the collection cap (E, F). In B-F, the dark circle is due to the laser engraving mark on the slide.

**Fig 2:**
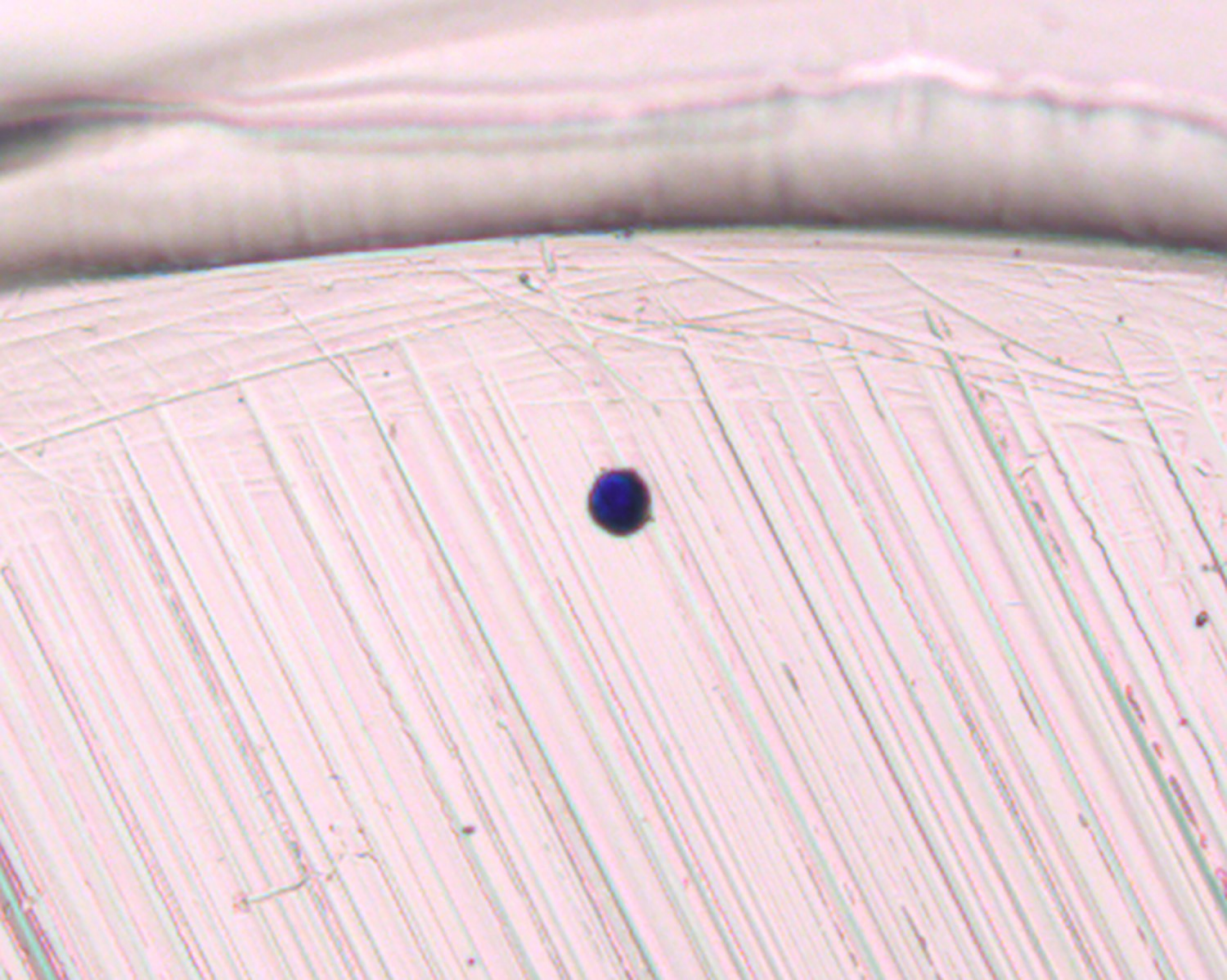
Visualization of SBS-microdissected cells in the tube collection cap. A Giemsa-stained microdissected nucleus cell, visualized at 63X magnification, is recovered in the 0.2 mL PCR tube collection cap.

The Leica Microsystems LMD6500 platform is optimized to microdissect cells from specific LMD membrane foil slides (Leica). To isolate cells from the SBS slides and limit the possible negative impact of the laser beam on cell nuclei, we set up appropriate laser parameters (power, aperture, speed, and specimen balance). A Full Factorial (2^4^) experimental plan, randomly replicated twice and considering three replicates of the center point, has been used to identify the best performing combination of laser parameters: power 32, aperture 15, speed 22, and specimen balance 0. The laser capture procedure of target cells is shown in Fig 1. These parameters, resulting in a fine cut at 63X magnification, allowed the isolation of single intact cells with efficiency in cell recovery close to 100% (Fig 2).

### 3.2 WGA and QC analyses indicated successful microdissection of intact single mimicking CTCs from cancer blood-like samples adhered on SBS slides

To test the method described here, we selectively microdissected single mimicking CTCs from the SBS slides prepared with mimic cancer patient blood samples, obtained using cancer cell lines spiked in WBCs from normal donors (see Materials and methods, and Table 1).

**Fig 3:**
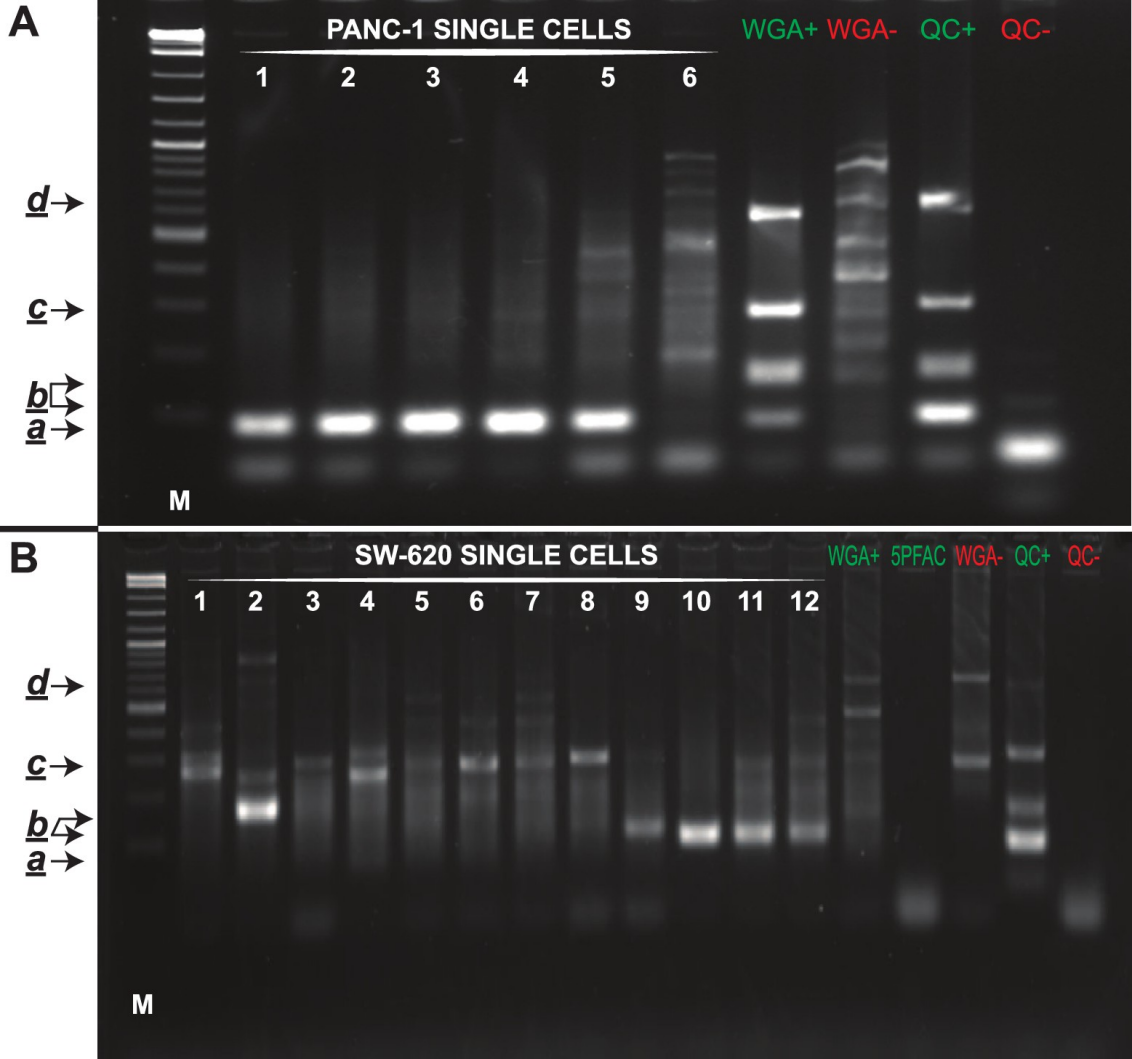
Examples of single-cell WGA QC analysis results. The gel electrophoresis shows examples of the QC analysis (consisting of multiplex PCR reactions, see paragraph 2.6) performed in cells microdissected from mimic cancer blood samples obtained with the PANC-1 (A) and SW-620 (B) tumour cell lines. WGA+ and WGA-: positive (30 pg of BEAS-2B genomic DNA) and negative controls of the WGA step, respectively; QC+ and QC-: positive (100 ng of BEAS-2B genomic DNA) and negative controls of the QC, respectively; 5PFAC: five BEAS-2B cells fixed in PFA 4%; M (molecular weight marker): 2-Log DNA ladder (New England Biolabs); NTC: no-template control. The black arrows pinpoint the size of the expected products, as reported in the Ampli1™ QC kit manufacturer’s instructions. All the samples showed at least one band except for sample 6 in (A).

Cancer cells were identified through an immunofluorescence assay previously developed for CTC identification [10], which is based on a positive staining for an epithelial marker (pan-cytokeratins) and negative staining for a hematopoietic marker (CD45). Once cancer cells have been identified, their coordinates are transferred to the microdissection platform for single-cell LCM. Following microdissection, the genomic DNA of each cell underwent WGA. The yield of single-cell WGA was comparable to positive controls, with an average value of 20 ng/μL (total sample volume 50 μL); a negligible DNA yield was observed for negative controls, demonstrating the absence of contamination during the procedure. The multiplex-PCR assay products obtained using the Ampli1™ QC kit, were analysed by gel electrophoresis (examples in Fig 3). In agreement with the Ampli1™ QC kit manufacturer’s instructions, samples with at least one of the four PCR products proved suitable for gene-specific assays, such as PCR and Sanger sequencing for mutation detection.

### 3.3 Mimicking CTC DNA analyses successfully identify cancer cell line specific mutations

After WGA and QC of microdissected single cells from cancer blood-like samples adhered on SBS slides obtained with the PANC-1 and SW-620 cell lines, we performed PCR with specific primer pairs to detect known point mutations (Table 1).

The results obtained are detailed in paragraphs 3.3.1–3.3.2.

#### 3.3.1 PANC-1

The PANC-1 cell line harbours a homozygous *TP53* c.818G>A (p.Arg273His) mutation (Table 1). Microdissected single cells isolated from cancer blood-like samples obtained with this cell line were tested for the presence of the mutation.

**Fig 4:**
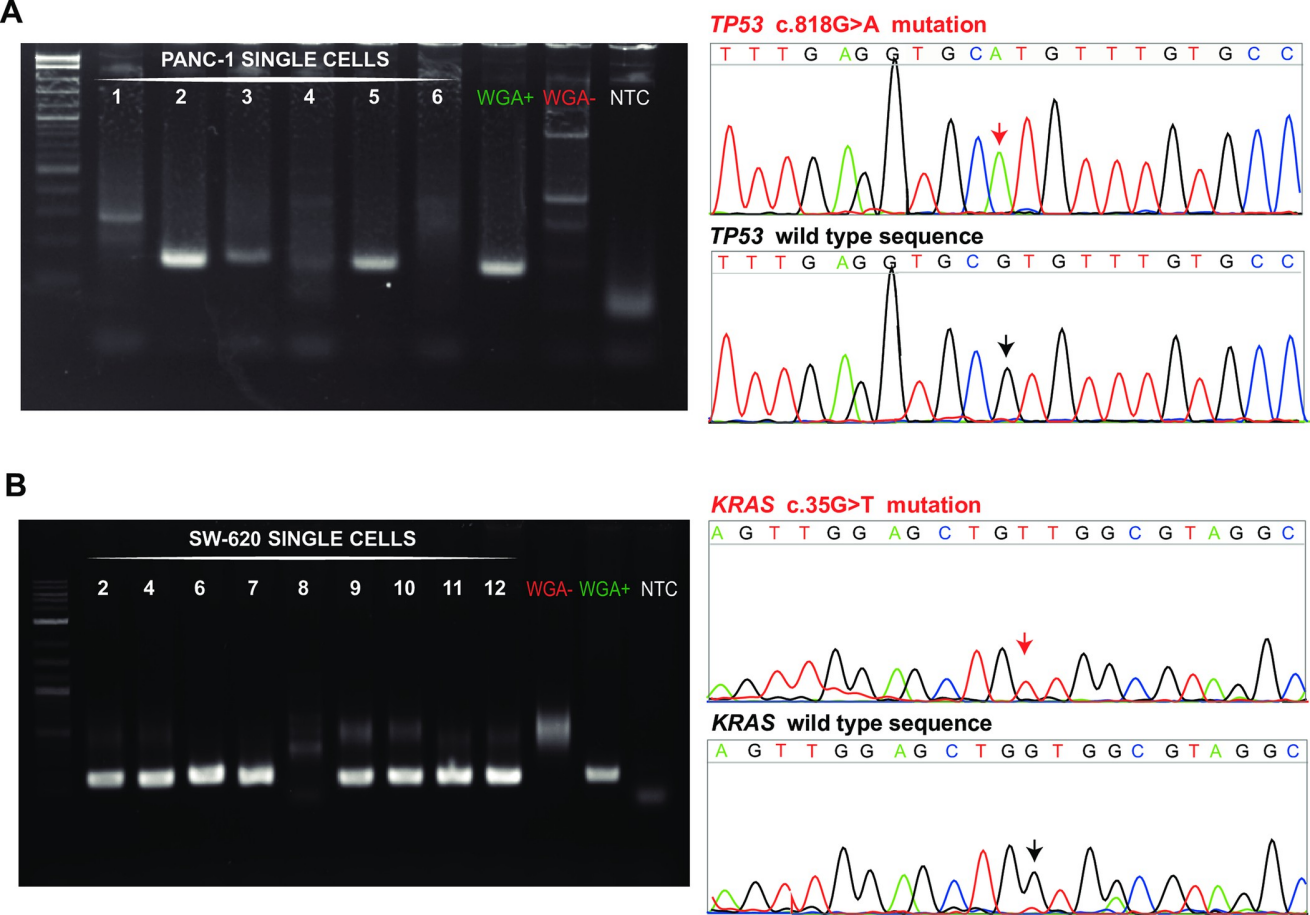
Detection of *TP53* and *KRAS* mutations in PANC-1 and SW-620 single-cell WGA products, respectively. PCR and Sanger sequencing chromatograms corresponding to the positive PCR products obtained using primers specific for *TP53* exon 4 (A) and *KRAS* exon 2 (B). WGA+ and WGA-: positive (30 pg of BEAS-2B genomic DNA) and negative controls of the WGA step, respectively; NTC: no-template control; M: 2-Log DNA ladder used as a DNA molecular weight marker.

After QC (Fig 3A), all WGA products were PCR amplified to detect the *TP53* mutation. The QC-negative sample did not show any PCR product (Fig 4A). Four out of five QC positive samples in Fig 3A resulted a band of the expected size (Table 2, Fig 4A), and Sanger sequencing analyses demonstrated they all corresponded to the *TP53* genomic segment harbouring the c.818G>A mutation (Fig 4A).

#### 3.3.2 SW-620

The SW-620 cell line harbours a homozygous *KRAS* c.35G>T mutation. QC analysis of single-cell DNA indicated samples of sufficient quality that were subsequently used for detecting the mutation by PCR and Sanger sequencing (Table 1). Only one out of nine QC-positive samples was negative to the PCR with *KRAS-*specific primers (Fig 3B). Sanger sequencing indicated the occurrence of the mutation in all samples (Fig 4B).

## 4. Discussion

Liquid biopsies are crucial diagnostic instruments for early, fast, and non-invasive cancer diagnosis. For this reason, the scientific community focuses on developing novel strategies for detecting and isolating CTCs.

We describe a method in which a Nanocoated slide, the SBS slide, that has been previously demonstrated to efficiently immobilize all white blood cell fractions derived from a blood sample collected in EDTA [10], has been used to isolate single cells using LCM and allowed for downstream genetic analysis.

The method described here shows several advantages compared with existing strategies, including the minimal manipulation of the sample, no loss of biological material (thanks to the use of SBS slides), and the easy molecular analysis at the single-cell level (e.g., DNA sequencing, PCR). After sample collecting, cells of interest, such as CTCs, are rapidly seeded on SBS slides and spontaneously adhere to their surface. Cells are then fixed with PFA, preserving their structure and morphology and minimizing changes in their behavior. Moreover, similarly to cell picking from slides, it is possible to preserve samples adhered to slides for retrospective studies [14].

The new method described here has made it possible to minimize the risk of losing biological material and identify cellular targets with a cell recovery index close to 100%. In this way, the main limitation of the CTC isolation techniques, being the low tumour cell number available for the analysis, has been overcome. Furthermore, the topography of the SBS slide surface mimicking the extracellular matrix potentially allows the adhesion of the whole range of CTC clones, as already described for the different WBC fractions [10]. This feature suggests that the heterogeneity of CTCs could be maintained after the adhesion on the SBS slides and analysed, providing a comprehensive snapshot of the original tumour.

Finally, our results reveal a good quality of the amplified CTC DNA, with the identification of the target mutations in 85% of the samples. This result is also due to the use of an Ultraviolet (UV) laser, which preserves the DNA integrity by preventing excessive material heating [15]. The UV light directly breaks the bonds between the molecules, resulting in photolytic degradation, which involves a higher absorption of the irradiated light by the material and, therefore, avoids using high powers to obtain microdissection [16].

## 5. Conclusions

The combined use of SBS slides and LMD could represent a quick and reproducible method to analyse single CTCs from liquid biopsies of cancer patients, leading to their identification by the single-cell screening of tumour-specific mutations. By integrating single-cell WGA, PCR, and Sanger sequencing, we detected cancer-specific point mutations. It has been previously demonstrated that processing of early breast cancer patient blood samples using SBS slides allowed 81% sensitivity for CTC identification in the luminal-like subtypes [10]. In addition, we reported a cell recovery index close to 100% and the identification of specific gene mutations in 85% of the analysed samples. Although further studies are needed to better specify the sensitivity range of our method in different tumour types, the data suggest that the combined use of SBS slides and LMD could be employed in a diagnostic workflow, ensuring high sensitivity.

Notably, the proposed method of recovery/identification of CTCs could be used for a broad range of downstream molecular applications, such as single-cell copy number variant analyses, whole genome sequencing or exome sequencing. Other applications, such as the analysis of circulating fetal cells for prenatal diagnosis, could also be envisaged.

## Supporting information

S1 File Step-by-step protocol, also available on protocols.io. (PDF)

S1 Raw images Original gel images for Figs 3 and 4. (PDF)
