# Supplementary material for: A novel method for the isolation of single cells mimicking circulating tumour cells adhered on Smart Bio Surface slides by Laser Capture Microdissection
Source: PLoS One. 2024 Mar 8;19(3):e0297739. doi: 10.1371/journal.pone.0297739 (PMC10923433; doi:10.1371/journal.pone.0297739)
Supplement: S1 File — (PDF) [file pone.0297739.s001.pdf]

A novel method for the isolation of single cells mimicking circulating tumour cells adhered on Smart Bio Surface slides by Laser Capture Microdissection

Grazia Visci[1], Doron Tolomeo[1], Angelo Lonoce[1], Aram Arshadi[1], Lorenzo Bascetta[2], Gianluca Trotta[3], Margot van Riel[4], Joris Robert Vermeesch[4], Roberta Carbone[2], Clelia Tiziana Storlazzi[1]

[1]Department of Biosciences, Biotechnology and Environment, University of Bari Aldo Moro, Bari, Italy;
[2]Tethis S.p.a., Milan, Italy;
[3]Institute of Intelligent Industrial Technologies and Systems for Advanced Manufacturing, Consiglio Nazionale delle Ricerche, Bari, Italy;
[4]Department of Human Genetics, KU Leuven, Leuven, Belgium

Roberta Carbone: Corresponding authors: roberta.carbone@tethis-lab.com (RC);
Clelia Tiziana Storlazzi: Corresponding authors: celiatiziana.storlazzi@uniba.it (CTS);

cleliatiziana.storlazzi

Protocol Info: Grazia Visci, Doron Tolomeo, Angelo Lonoce, Aram Arshadi, Lorenzo Bascetta, Gianluca Trotta, Margot van Riel, Joris Robert Vermeesch, Roberta Carbone, Clelia Tiziana Storlazzi . A novel method for the isolation of single cells mimicking circulating tumour cells adhered on Smart Bio Surface slides by Laser Capture Microdissection. **protocols.io** https://protocols.io/view/a-novel-method-for-the-isolation-of-single-cells-m-c3szynf6

Created: Sep 27, 2023

Last Modified: Oct 25, 2023

PROTOCOL integer ID: 89657

Funders Acknowledgement:
Programma Operativo Nazionale FSE-FESR Ricerca e Innovazione 2014-2020, Azione I.1 “Dottorati innovativi a caratterizzazione industriale”
Grant ID: DOT1302377 (CUP: H92H18000030006) supporting G.V.

ABSTRACT

In recent years, the importance of isolating single cells from blood circulation for several applications, such as non-invasive tumour diagnosis, the monitoring of minimal residual disease, and the analysis of circulating fetal cells for prenatal diagnosis, urged the need to set up innovative methods for such application. Different methods were developed. All show some weaknesses, especially a limited sensitivity, and specificity. Here we present a new method for isolating a single or a limited number of cells adhered to SBS slides (Tethis S.p.a.) (a glass slide coated with Nanostructured Titanium Dioxide) by Laser Capture Microdissection and subsequent Whole Genome Amplification. SBS slides have been shown to have an optimal performance in immobilizing CTC from early breast cancer patients. In this work, we spiked cancer cells in blood samples to mimic CTCs. By defining laser parameters to cut intact samples, we were able to isolate genetically intact single cells. We demonstrate that SBS slides are optimally suited for isolating cells using LCM and that this method provides high-quality DNA, ideal for gene-specific assays such as PCR and Sanger sequencing for mutation analysis.

ATTACHMENTS

854-2210.docx

INTRODUCTION

Genomic instability is a hallmark of cancer cells [1], implying the occurrence of various structural and numerical genetic mutations and resulting in the genesis of diverse tumour cell sub-populations (tumour heterogeneity) [2,3]. A portion of cancer cells is shed from the primary solid tumour into the blood vessels after epithelial-mesenchymal transition (EMT) resulting in circulating tumour cells (CTCs). Those cells can survive in the peripheral vascular system and initiate the development of metastases. Because of their essential role in the metastatic process, CTCs have been investigated as diagnostic, prognostic, and predictive biomarkers in many types of cancer. They have been used to enable early detection of tumour lesions (primary and metastatic), to identify minimal residual disease and to stratify patients, based on the detection of therapeutic targets or resistance mechanisms [4].

Cancer cell heterogeneity represents a significant challenge for targeted therapies in the clinical management of patients [5]. In this context, the isolation of CTCs is crucial for the non-invasive molecular analysis of cancer cells in patients.

To date, several technologies have been developed to isolate and characterize CTCs from blood. Those methods are usually based on the enrichment and/or depletion of CTCs using specific antibodies, filtration by size, or separation by microfluidic systems [2]. These methods lay their foundations on the biological and physical features of CTCs [5]. However, CTCs isolation remains a major challenge because of two main issues: i) CTCs are extremely rare in the blood vessels (0-1,000 CTCs among 5 billion erythrocytes and 10 million leukocytes per mL of blood) [6]; ii) the detection of CTCs is technically demanding. In principle, a perfect approach would imply the analysis of an “unmanipulated” clinical sample to minimize the risk of CTC loss.

The last years have seen considerable innovation in instrumentation and analytical techniques for single-cell isolation. One of these is the Laser Capture Microdissection (LCM) technology [7]. LCM is an ideal tool for rapidly collecting a large number of single cells. It can precisely target and capture cells of interest, despite tissue heterogeneity. LCM contact-free isolation and separation allows precision and avoid contamination [8]. This method is quick and versatile, used in various applications, including the molecular characterization of cancer cells. Also, it offers the possibility of identifying specific cells by labelling them with fluorescent antibodies [9].

The present work describes a new strategy for isolating and analysing single cells mimicking CTCs by combining the LCM technology with Whole Genome Amplification (WGA). Smart BioSurface (SBS) slides were used to optimize the yield of CTC recovery from blood. These slides are capable to efficiently immobilize living and non-spontaneously adhering cells, such as cells deriving from blood, enabling a comprehensive capture approach to liquid biopsy [10]. Cluster-assembled TiO2 films result from a random stacking of nanoparticles and are characterized, at the nanoscale, by granularity and porosity mimicking extracellular matrix structures [11]. Previous results indicated that cluster-assembled nanostructured TiO2 is a biocompatible surface for the cell adhesion of different kinds of normal and cancer

cells [12].
To validate our method, we mimicked CTCs in liquid biopsy-like samples by mixing cancer cell line cells with white blood cells (WBCs) from healthy donors. We chose two cancer cell lines (PANC-1 and SW-620) as representatives of the most devastating tumour types (pancreas and colon carcinoma, respectively) and focused on specific hotspot mutations in frequently mutated genes (Table 1). The samples were dispensed on SBS slides, and the cancer cells, identified by immuno-staining, were selectively isolated by LCM. We obtained a high-efficiency recovery of single cancer cells by LCM and an effective amplification of single-cell DNA by Whole Genome Amplification, as evaluated by its Quality Control. The efficacy of the method consists of the possibility to perform downstream molecular analyses at the single-cell level, such as identifying specific nucleotide mutations. Hence, the present methodology could be offered as a potential non-invasive approach for identifying and capturing CTCs from the blood of cancer patients. Interestingly, the present methodology could also be applied for other purposes, such as isolating circulating fetal cells for non-invasive prenatal diagnosis.

Table 1. Cell lines and mutations investigated to validate the proposed cell isolation method.

A	B	C
**Cell line**	**Nucleotide mutation**	**Zygosity status**
PANC-1	TP53 c.818G>A (p.Arg273His)	Homozygous
SW-620	KRAS 35G>A (G12D)	Homozygous

Acknowledgements

We thank Dr. Giulia Daniele for technical assistance in the setup of the microdissection procedure.

Author contributions

GV and DT isolated the mimicking CTCs by LCM; MVR and JRV supervised and planned WGA assays; GV, DT, and AL performed WGA and Quality control experiments. AA carried out PCR validations; LB cultured the cells, prepared the spiked-in samples, and performed the immune-staining assays. GT set the laser parameters. CTS, RC, DT, and GV analyzed data and wrote the manuscripts. RC and CTS designed the research. All the authors revised the manuscript.

Materials

- NH_4Cl
- $KHCO_3$
- EDTA
- DPBS (Sigma-Aldrich)
- Trypan blue (Sigma-Aldrich)
- paraformaldehyde (PFA) (Sigma-Aldrich)
- DMEM medium (EuroClone, Pero, Milan, Italy)
- RPMI-1640 medium (EuroClone)
- 10% fetal bovine serum (EuroClone)
- Penicillin-Streptomycin mixture (Sigma-Aldrich)
- gSYNCTM DNA Extraction Kit (Geneaid Biotech Ltd., New Taipei City, Taiwan)
- Qubit™ Fluorometer (Life Technologies, Carlsbad, USA)
- methanol (Sigma-Aldrich)
- Normal Goat Serum (Cell Signalling, Danvers Massachusetts, United States, product code: 5425)
- anti-CD45 pan-leukocyte marker (Bio-Rad Laboratories, Inc., CA, USA, product code: MCA87)
- anti-pan-cytokeratins antibody (Sigma-Aldrich, product code: C2562)
- Hoechst (ThermoFisher Scientific, Waltham, Massachusetts, USA)
- ZEISS AxioImagerZ2 microscope
- Giemsa stain (ThermoFisher Scientific)
- Leica Microsystems LMD6500 platform (Leica Biosystems, Wetzlar, Germany)
- 0.5% NP40
- Proteinase K
- Tris-HCl pH 8
- Ampli1™ WGA Kit (Menarini Silicon Biosystems, Castel Maggiore, Italy)
- Ampli1™ QC kit (Menarini Silicon Biosystems)
- 2% agarose, Condalab, Madrid, Spain
- GelRed™ (Biotium, Landing Pkwy, Fremont, USA)
- ChemiDocTM MP Imaging System (Bio-Rad Laboratories)

Preparation of spiked-in samples

5m

1 Collect healthy donor blood, after discarding the first 3 mL of the blood draw to minimize keratinocyte contamination.

2 Process blood samples with red blood cell lysis through ammonium chloride buffer.

Ammonium chloride buffer

A	B
NH_4Cl	0.15 M
$KHCO_3$	9.93 mM

A	B
EDTA	0.13 mM

3 Then separate WBCs by centrifugation Centrifuge 400 rpm, Room temperature, 00:05:00, resuspend in DPBS, and count using Trypan blue.

4 Then spike a predefined number of cells (50-100 cells for each slide) from selected cancer cell lines (see Cell lines selection and culturing) into WBCs pellet.

5 Dispense the cell mix on SBS slides, patented by Tethis S.p.A (Milan, Italy), at the optimal concentration of 2.5 x 10^6 cells per slide.

6 Subsequently, fix the slides with 4% paraformaldehyde (PFA) and store at Temperature -80 °C.

Cell lines selection and culturing

7 To validate the method, choose a cancer cell line sample harbouring a known point mutation.

8 Choose as a WGA positive control a high-quality extracted DNA.

9 Culture the selected cell line in the appropriate culture conditions.

10 Incubate all cells at 37 °C and 5% CO_2.

11 For spike-in and WGA experiments, gently detach cells, resuspend in DPBS, and count with Trypan blue.

Mimicking CTC detection by Immuno-staining

12 Prepare slides with samples obtained by mixing cell line cells with WBCs. Then, permeabilize it with methanol ice-cold and block it in a solution of 5% Normal Goat Serum.

13 Perform immunostaining with an anti-CD45 pan-leukocyte marker and an anti-pan-cytokeratins antibody.

14 Counterstain nuclei with Hoechst.

15 After staining, scan slides through an automated fluorescence microscope and analyze using Tethis proprietary imaging algorithms.

16 Trained operators manually select cancer cell line cells (mimicking CTCs) by following the criteria of staining positivity to pan-CKs and negativity to CD45.

17 Also, identify the position of each mimicking CTC using slide coordinates, which will be appropriately transferred to the LCM microscope.

Giemsa staining

25m

18 Soak the slide in a Coplin jar containing PBS 1X for 00:05:00 to facilitate the cover slide removal. 5m

19 After gently removing the cover slide, air dry the slide in a vertical position and stain by adding 1 mL of 7.4% Giemsa stain for 00:20:00 . 20m

20 Then, wash it by briefly dipping in and out of a Coplin jar containing distilled water (one or two dips).

21 Finally, air dry the slide vertically and observe it under the microscope.

Isolation of mimicking CTCs by LCM

30m

22 Carry out the LCM setting up appropriate laser parameters.

Note

We searched for the mimicking CTCs at a 10× magnification by exploiting the previously identified slide coordinates (as described in **Mimicking CTC detection by Immuno-staining**).

23 Perform the LCM at a 63X magnification.

23.1 First, microdissect the WBCs around the mimicking CTC into a 'trash' collection cap of a 0.2 mL DNase-free PCR tube.

23.2 Subsequently, microdissect the single cell of interest into a new cap filled with 30 µL of a Lysis Buffer to prevent the risk of sample loss.

Lysis Buffer

A	B
NP40	0.50%

A	B
Proteinase K	0.67 µg/µL
Tris-HCl pH 8	0.032 M

24 After visualizing the single cell into the cap, unload the tube holder, carefully recover the PCR tube, and close it.

25 For negative controls, add 30 µL of Lysis Buffer to an empty PCR tube cap.

26 Incubate the collected samples at 50 °C Overnight to allow the cells to lysis (without overturning the tubes). 20m

27 After the incubation, spin down the samples in a centrifuge for 00:10:00 at maximum speed and store at 4 °C until further processing. 10m

WGA and Quality control (QC)

1h 20m

28 Perform DNA amplification using the Ampli1TM WGA Kit since it allows genome amplification from single fixed cells, as reported in the manufacturer's instructions.

29 Since the WGA requires a minimal sample volume of 1 µL, let the samples evaporate using a heater at 50 °C, then proceed with WGA, following the manufacturer's instructions.

30 Briefly, samples undergo a second lysis step with a 2 µL lysis reaction mix (42 °C, Overnight). 10m

31 Digest DNA by adding 2 µL of digestion reaction mix and incubating at 37 °C for 00:05:00 . 5m

32 Following enzyme inactivation (65 °C for 00:05:00), add 5 µL of ligation mix, and ligate the pre-annealed adapter nucleotides to the fragmented DNA at 15 °C for 01:00:00 . 1h 5m

33 Finally, add the Primary PCR Reaction Mix 40 µL to each sample to obtain the DNA amplification.

34 To assess the performance of the Ampli1™ method, each reaction run should include negative and positive controls. Add the negative (not template) control, consisting of Amount 1 µL of H2O, to exclude possible contamination during WGA.

35 Use 30 pg of high-quality DNA as a positive control to test the performance of our method.

36 Measure DNA concentration of the WGA products using the Qubit™ Fluorometer.

37 Check the quality of WGA products by performing a multiplex-PCR using primer pairs amplifying three/four PCR products of different size (100-600 bp).

> **Note**
>
> PCR products with at least one/two out of four bands are suitable for gene-specific analyses (e.g., for detecting specific point mutations).
> Three/four PCR products indicate that the samples could be used for successful genome-wide studies (metaphase/array CGH)

38 Check the samples by agarose gel electrophoresis (2% agarose).

Polymerase chain reaction (PCR) and Sanger sequencing

39 Perform PCR assays to specifically amplify genetic regions harbouring the selected point mutations in each cell line used to validate the method.

40 Design the primer pairs to detect the target mutation.

41 Perform PCR experiments, Sanger sequencing, and sequence analyses.
