# Supplementary material for: A novel method for the isolation of single cells mimicking circulating tumour cells adhered on Smart Bio Surface slides by Laser Capture Microdissection
Source: PLoS One. 2024 Mar 8;19(3):e0297739. doi: 10.1371/journal.pone.0297739 (PMC10923433; doi:10.1371/journal.pone.0297739)

FIGURE 3A (gel image obtained by using the ChemiDoc™ MP Imaging System, Bio-Rad Laboratories)

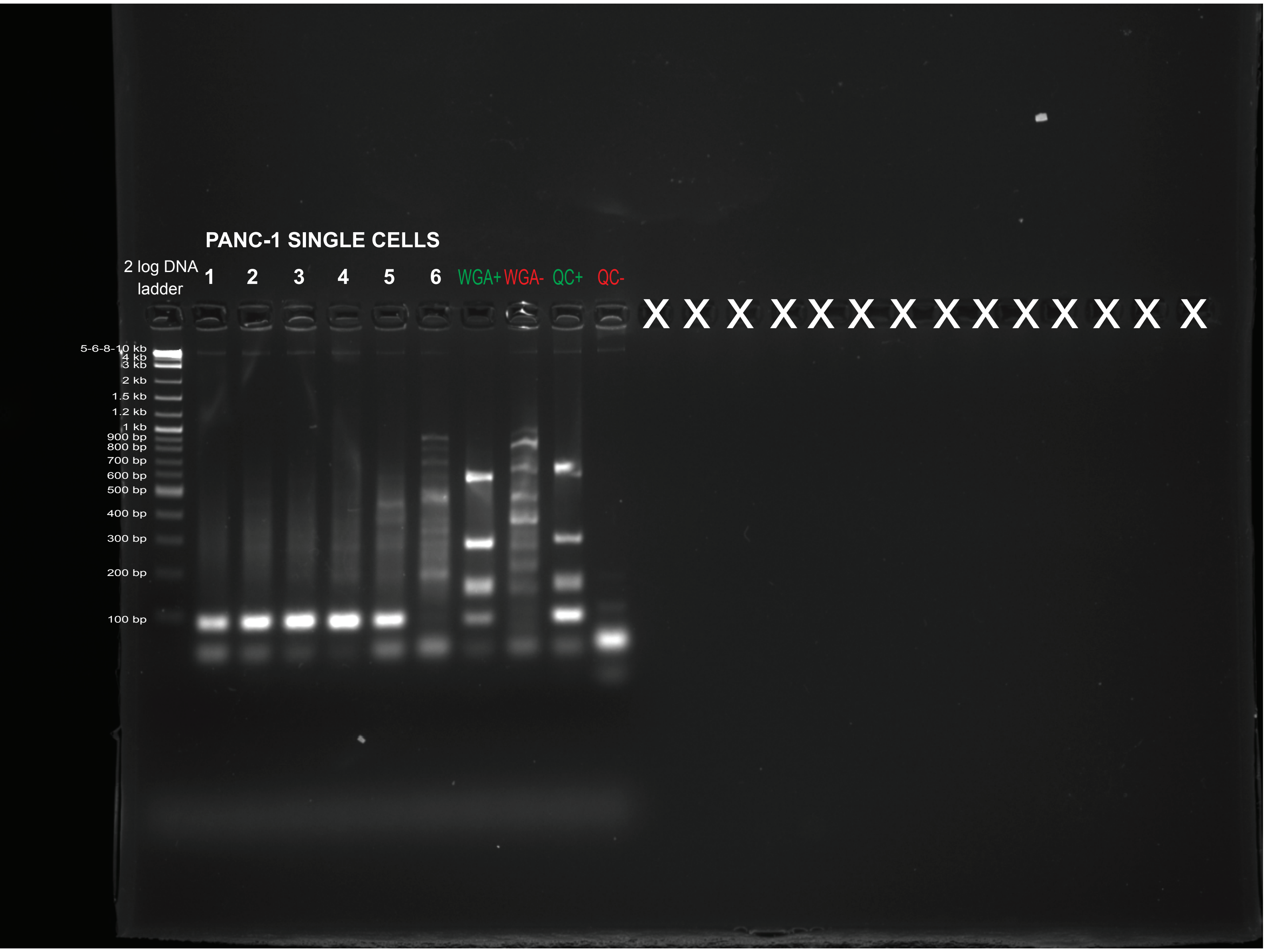

FIGURE 3B (gel image obtained by using the ChemiDoc™ MP Imaging System, Bio-Rad Laboratories)

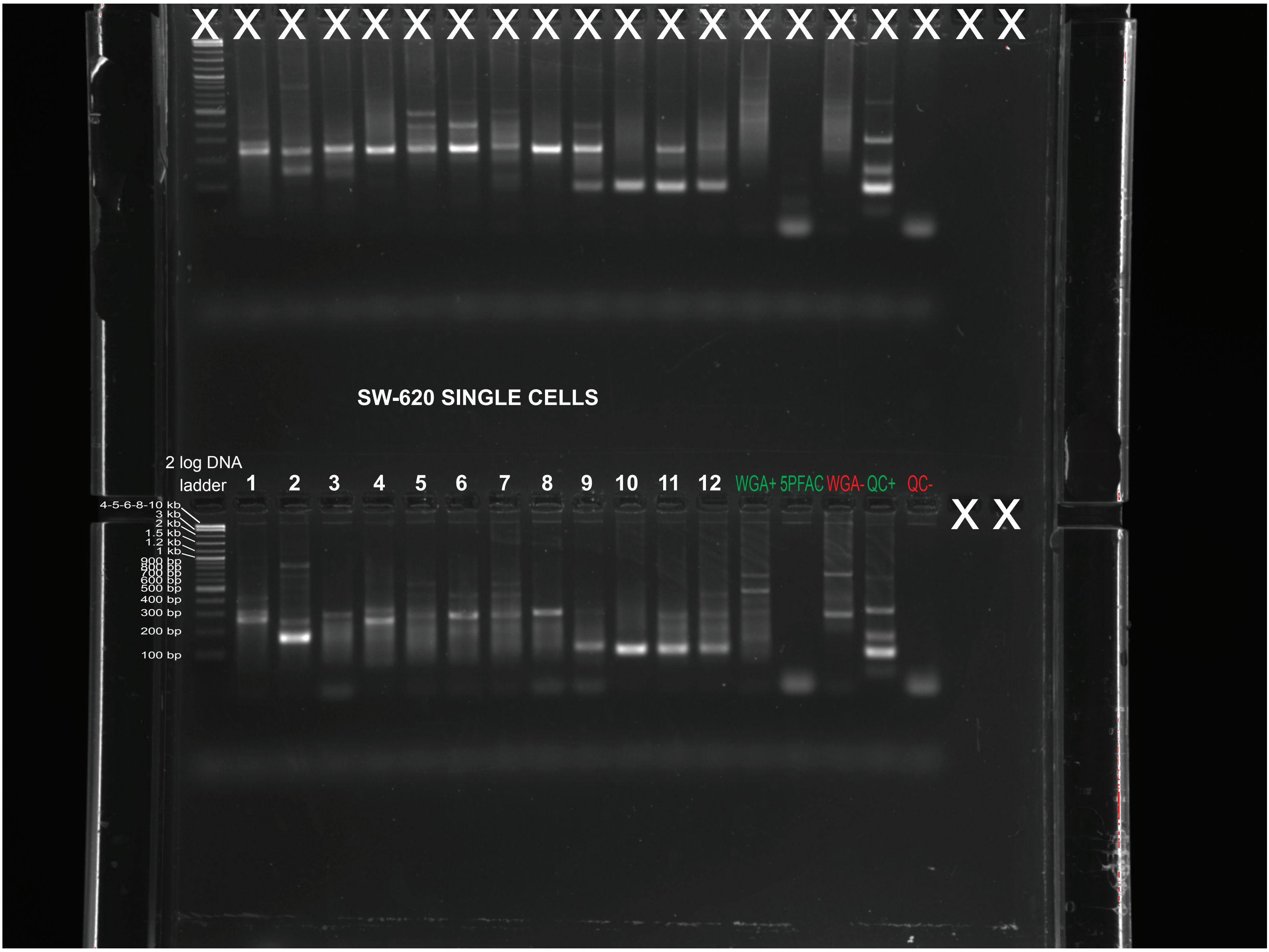

FIGURE 4A (gel image obtained by using the ChemiDoc™ MP Imaging System, Bio-Rad Laboratories)

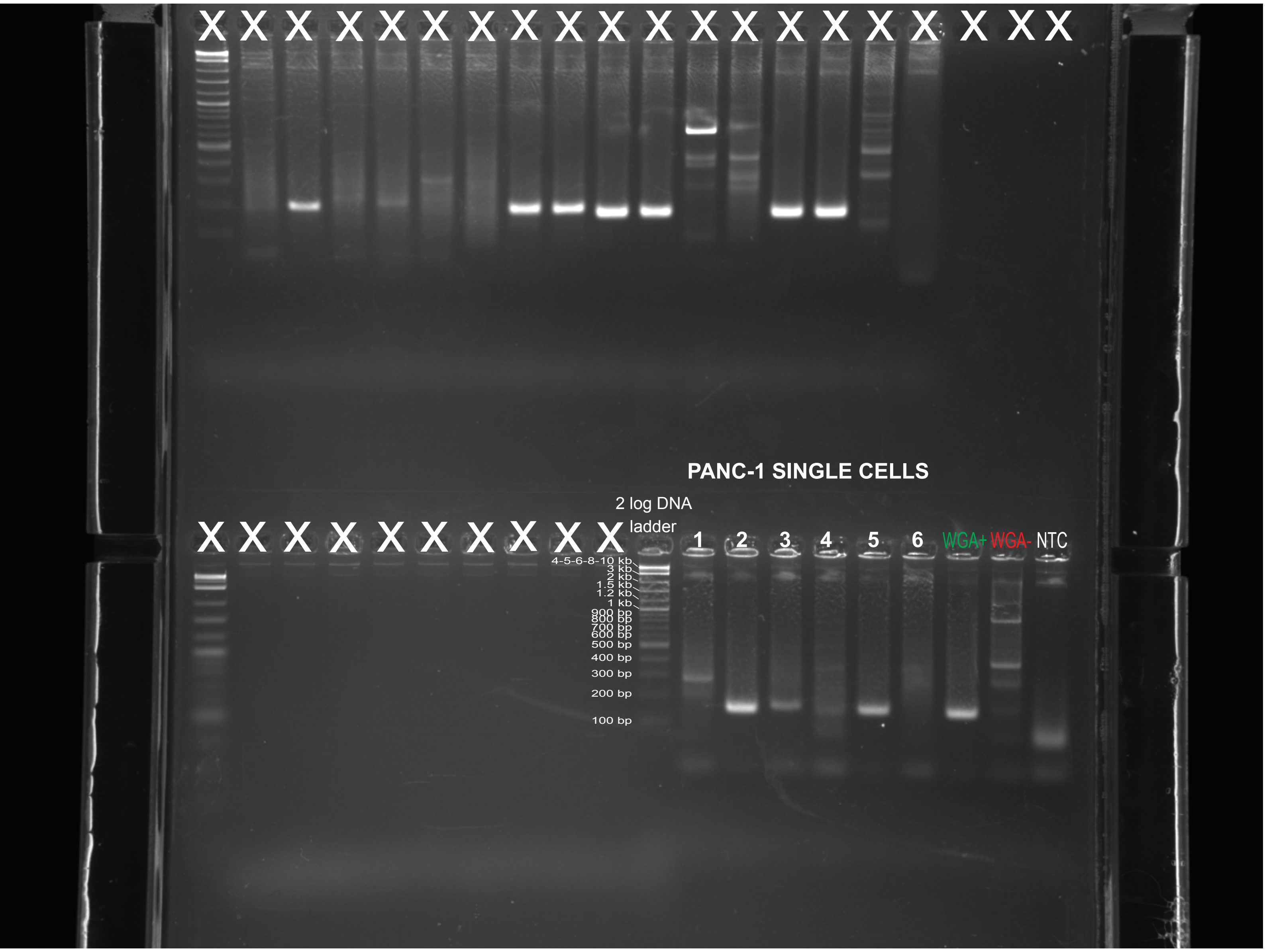

FIGURE 4B (gel image obtained by using the ChemiDoc™ MP Imaging System, Bio-Rad Laboratories)

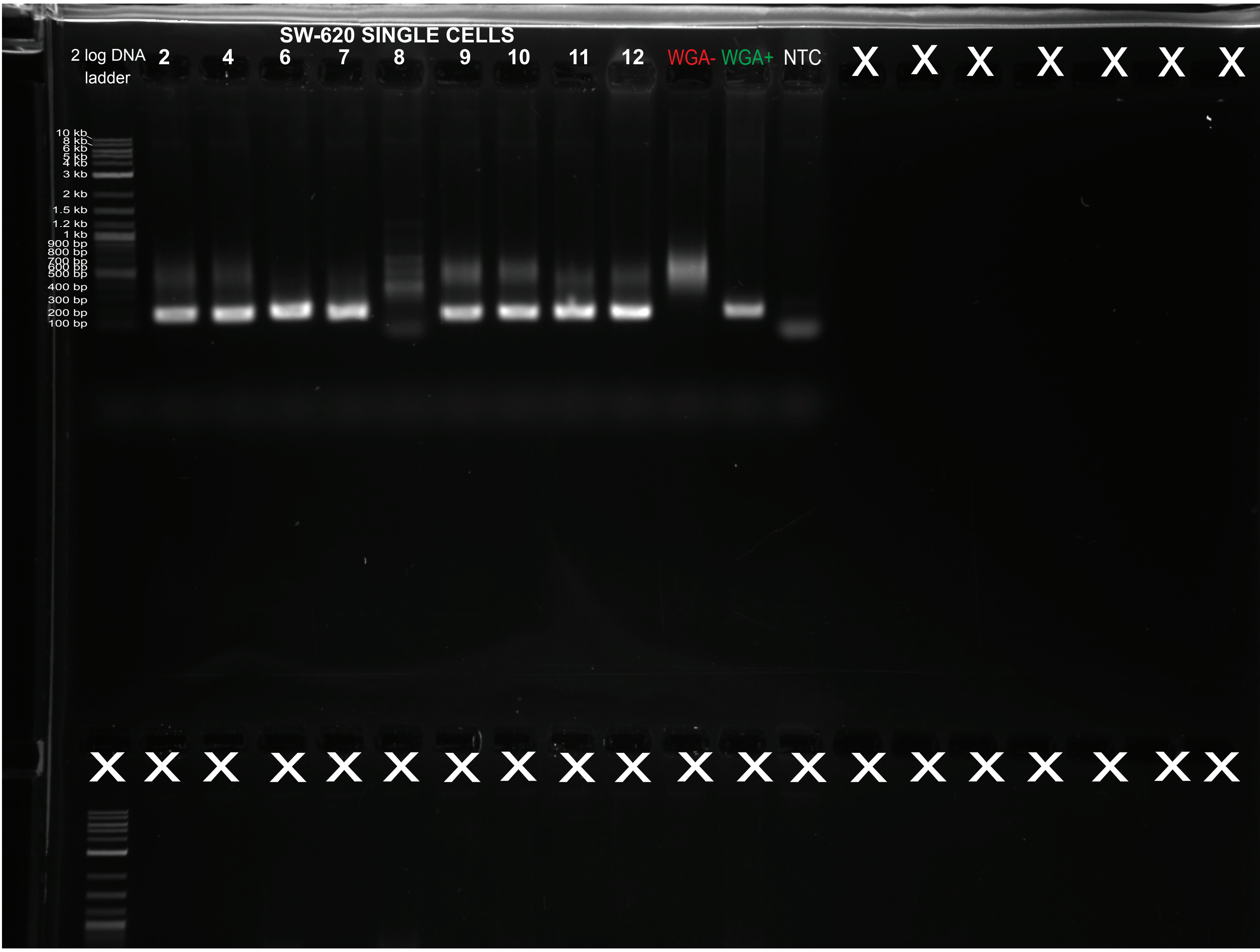

Supplement: S1 Raw images — (PDF) [file pone.0297739.s002.pdf]
